# At-home specimen self-collection as an additional testing strategy for chlamydia and gonorrhoea: a systematic literature review and meta-analysis

**DOI:** 10.1136/bmjgh-2024-015349

**Published:** 2024-08-27

**Authors:** Amanda C Smith, Phoebe G Thorpe, Emily R Learner, Eboni T Galloway, Ellen N Kersh

**Affiliations:** 1 Division of STD Prevention, Centers for Disease Control and Prevention, Atlanta, Georgia, USA

**Keywords:** Systematic review, Diagnostics and tools, Infections, diseases, disorders, injuries, Public Health

## Abstract

**ABSTRACT:**

**Introduction:**

*Chlamydia trachomatis* (Ct) and *Neisseria gonorrhoeae* (Ng) infections are often asymptomatic; screening increases early detection and prevents disease, sequelae and further spread. To increase Ct and Ng testing, several countries have implemented specimen self-collection outside a clinical setting. While specimen self-collection at home is highly acceptable to patients and as accurate as specimens collected by healthcare providers, this strategy is new or not being used in some countries. To understand how offering at home specimen self-collection will affect testing uptake, test results, diagnosis and linkage to care, when compared with collection in clinical settings, we conducted a systematic literature review and meta-analysis of peer-reviewed studies.

**Methods:**

We searched Medline, Embase, Global Health, Cochrane Library, CINAHL (EBSCOHost), Scopus and Clinical Trials. Studies were included if they directly compared specimens self-collected at home or in other non-clinical settings to specimen collection at a healthcare facility (self or clinician) for Ct and/or Ng testing and evaluated the following outcomes: uptake in testing, linkage to care, and concordance (agreement) between the two settings for the same individuals. Risk of bias (RoB) was assessed using Cochrane Risk of Bias (RoB2) tool for randomised control trials (RCTs).

**Results:**

19 studies, from 1998 to 2024, comprising 15 RCTs with a total of 62 369 participants and four concordance studies with 906 participants were included. Uptake of Ct or Ng testing was 2.61 times higher at home compared with clinical settings. There was a high concordance between specimens collected at home and in clinical settings, and linkage to care was not significantly different between the two settings (prevalence ratio 0.96 (95% CI 0.91–1.01)).

**Conclusion:**

Our meta-analysis and systematic literature review show that offering self-collection of specimens at home or in other non-clinical settings could be used as an additional strategy to increase sexually transmitted infection testing in countries that have not yet widely adopted this collection method.

**WHAT IS ALREADY KNOWN ON THIS TOPIC**

**WHAT THIS STUDY ADDS**

- In various settings and populations, specimen self-collection at home or other non-clinical settings led to increases in testing, which could be crucial in ensuring follow-up testing after treatment and for partners of infected individuals.
- There was no difference in linkage to care between those providing specimens collected at home compared with those who collected specimens in clinical settings.

**HOW THIS STUDY MIGHT AFFECT RESEARCH, PRACTICE OR POLICY**

- Expanding opportunities for specimen self-collection at home or in other non-clinical settings could be a viable way to increase accessibility to testing, health equity and limit further spread of Ct and Ng.

## Introduction

### Description of the infections

 Chlamydia and gonorrhoea are the leading reported bacterial sexually transmitted infections (STIs) worldwide with an estimated 211 million combined cases in 2020, according to the WHO.^1^ Many of these infections are asymptomatic, making screening crucial for detection, treatment and prevention of sequelae in both individuals and their partners. Over the past decade, reported cases of chlamydia and gonorrhoea have risen in many countries with established screening programmes.^2^,^6^ However, these reports likely underestimate the true number of infected individuals.

Several countries including the USA,^7^ Canada^8^ and England^9^ have annual screening recommendations for chlamydia and/or gonorrhoea for sexually active populations at higher-risk for acquiring STIs. These populations include, but are not limited to, young women and adolescents (under 25), pregnant individuals and men who have sex with men (MSM). Despite these recommendations, less than 62% of women under 25 in the USA^10^ and less than 21% in England^11^ participate in screening. This is particularly concerning as untreated *Chlamydia trachomatis* (Ct) and *Neisseria gonorrhoeae* (Ng) infections can lead to serious health complications such as pelvic inflammatory disease (PID), infertility and ectopic pregnancy.^12 13^ Annual Ct and Ng screening at sites of exposure is recommended for MSM by the CDC and other agencies, with more frequent testing recommended for MSM on HIV pre-exposure prophylaxis (PrEP).^14^ In 2017, only 42% of MSM reported being screened for Ct and Ng and only 16% reported extragenital testing in the past 12 months.^15^ Recent studies suggest that STI screening in MSM reduces chlamydia incidence by 15%,^16^ and increased screening for MSM taking PrEP would result in a 17% decrease in asymptomatic STIs. Therefore, strategies to boost screening, especially among young women and MSM, should be fully explored.

### Description of the intervention

Nucleic acid amplification tests (NAATs) are recommended for the screening and diagnosis of both Ct and Ng due to their high sensitivity and specificity.^14 17 18^ Variations of NAATs include ligase chain reaction, transcription-mediated amplification, PCR and strand displacement amplification. NAATs can be used with a variety of specimen types. However, self-collected vaginal swabs from females and urine from males are the most sensitive for detecting urogenital Ct and Ng infections.^17 19^ If extragenital testing is needed, NAATs approved for use with extragenital specimens are also highly sensitive and specific.^11^ To increase screening, self-collection of specimens should be considered. Patients report that self-collection of vaginal swabs and rectal swabs in a clinical setting for Ct and Ng is preferred over healthcare provider (HCP)-collection because it offers increased patient autonomy, confidentiality and convenience.^20^,^22^ In addition to the increased acceptability, self-collection of vaginal specimens and extragenital specimens can be more accurate than HCP-collected specimens.^23^,^28^ As such, both the WHO and CDC recommend self-collection of vaginal swabs, first-void urine, pharyngeal, rectal and urethral swabs be available for Ct and Ng testing.^17 29^

Specimen self-collection at home or in other non-clinical settings refers to the process of collecting a sample, such as a vaginal swab or urine, at home or anywhere outside a healthcare facility without direct clinical supervision. The specimens are then mailed to a testing laboratory or dropped off at a healthcare facility or other drop-off location. This method can reduce logistical barriers like transportation issues and limited clinic availability and is useful for those who want private or more frequent testing, have had negative experiences with HCPs, or prefer greater control over their healthcare. During the COVID-19 pandemic, demand for self-testing and self-collection kits grew.^30^,^33^ When England’s National Chlamydia Screening Programme began offering internet-based testing in 2015, only ~5% of tests were conducted online; by 2022, 43% were.^11^ Internet-based testing programmes vary; some kits can be requested by a patients or providers, with some services offering direct treatment following a positive test result, while others link patients to local HCPs for follow-up testing and treatment.^30^,^34^ The cost of the self-collection kits also varies widely, depending on whether the service is privately-funded or government-funded.^30^,^33^

Prior systematic literature reviews evaluated at-home specimen self-collection compared with collection (either self or clinician) in a healthcare facility for Ct/Ng.^35 36^ In 2015, Fajardo-Bernal and colleagues concluded there was no significant difference between home self-collection and collection in a healthcare facility for a number of testers and positive tests,^36^ and Odesanmi *et al* suggested that home self-collection increased testing in women.^35^ Since these reviews were conducted, advances include more sensitive NAATs, new near point-of-care tests for Ct and Ng, new evidence on the acceptability of home self-collection, and increasing demand for autonomy and acceptance of self-collection. Therefore, we performed a current systematic literature review and meta-analysis to assess whether specimen self-collection at home or in other non-clinical settings for Ct and Ng testing increases testing uptake, Ct or Ng diagnosis, linkage to care and has high concordance compared with collection in a healthcare facility.

## Methods

### Research question and inclusion criteria

We aimed to address whether specimen self-collection at home for Ct and Ng testing should be offered as an additional approach to specimen collection in clinical settings.

### Population

All sexually active individuals who were recruited to participate in the study by any method. We did not place any age restrictions for the studies included in our analysis, but some studies had specific age restrictions as indicated in table 1.

**Table 1:** Table of evidence

Study, location and population	Intervention	Study methods	Key outcomes
*Study*: Anderson 1998 *Location*: Aarhus, Denmark *Population*: male sexual partners of Ct-positive women attending a general practice clinic	*Intervention*: index women supplied male sexual partners with an envelope containing a 10 mL sterile container, information on collecting the first urine sample and a prepaid envelope for returning the sample *Control*: index women supplied male sexual partners with envelopes which contained a request for the partner to visit his doctor as well as a contact slip and a prepaid envelope to be given to the doctor for returning urethral swab	*Study design*: RCT *Sample size*: n=133 Intervention: n=65 Control: n=68 *Specimens tested*: Intervention: urine Control: urethral *Diagnostic test*: Amplicor, Roche *Primary endpoint*: 6 months	Higher proportion of people assigned to the intervention group tested for Ct (44/65) compared with the control group (19/68) PR=2.42 (1.60–3.68) High number of Ct cases diagnosed in the intervention group (12/65) compared with the control group (7/68) PR=1.79 (0.75–4.27)
*Study*: Ostergaard 1998 *Location*: Aarhus, Denmark *Population*: high-school age, sexually active men and women in Aarhus County	*Intervention*: females were asked to collect two urine samples and one vaginal flush sample and the males were asked to collect one first void urine sample at home. These samples were mailed directly to the microbiology dept. for testing. Asked to give address for receipt of test results *Control*: students were offered testing at their doctors or at the local clinic for STIs	*Study design*: RCT *Sample size*: men: n=688, women: n=1761 Intervention: men: n=442, women: n=928 Control: men: n=246, women: n=833 *Specimens tested*: men: urine, women: vaginal flush and urine *Diagnostic test*: PCR *Primary endpoint*: not stated	Higher proportion of men and women assigned to the intervention group tested for Ct (men=430/442, women=867/928) compared with the control group (men=4/246, women=63/833) PR=men: 59.83 (22.63–158.16), women: 12.35 (9.74–15.67) High proportion of Ct cases in the intervention group (men: 11/442, women: 43/928) compared with the control group (men: 1/246, women: 5/833) PR=men: 6.12 (0.8–47.14), women: 7.72 (3.07–19.40)
*Study*: Ostergaard 2003 *Location*: Denmark *Population*: male and female sexual partners of Ct-positive men and women	*Intervention*: index patients were asked to supply their sexual partners with a collection kit. For male partners, the kit contained a 10 mL tube for a first void urine sample and for female partners the kit consisted of a vaginal pipette containing 5 mL sterile normal saline *Control*: index patients supplied sexual partners with envelopes which contained a request for the partner to visit his doctor and the collection kit to bring to the visit which was identical to the home collection kit	*Study design*: RCT *Sample size*: men: n=631, women: n=103 Intervention: men: n=342, women: n=56 Control: men: n=289, women: n=47 *Specimens tested*: men: urine, women: vaginal flush *Diagnostic test*: LCx CT test *Primary endpoint*: not stated	Higher proportion of men and women assigned to the intervention group tested for Ct (men=195/342, women=38/56) compared with the control group (men=88/289, women=9/47) PR=men: 3.54 (1.92–6.55), women: 1.87 (1.54–3.68) High proportion of Ct cases in the intervention group (men=74/342, women=17/56) compared with the control group (men=45/289, women=5/47) PR=men: 1.39 (0.99–1.94), women: 2.85 (1.14–7.15)
*Study*: Cook 2007 *Location*: Western Pennsylvania, USA *Population*: sexually active young women (15–24) at higher risk for acquiring STIs	*Intervention*: participants in the intervention group were directly mailed a test kit for Ct and Ng. Women received a testing kit at 6, 12 and 18 months after enrolment. The testing kit materials included a cover letter, an instruction sheet, brief questionnaire, vaginal swab, prelabelled swab container and postage-paid mailing carton *Control*: women assigned to the control group received a postcard at 6, 12 and 18 months after enrolment. The postcard included information similar to the cover letter provided with the home test kits, and participants were invited to attend one of the participating study clinics for a routine test for women’s health infections at no cost	*Study design*: RCT *Sample size*: n=420 Intervention: n=211 Control: n=209 *Specimens tested*: vaginal swab *Diagnostic test*: BD ProbeTec *Primary endpoint*: 18 months	Higher proportion of women assigned to the intervention group tested for Ct and Ng (162/211) compared with the control group (117/209) PR=1.37 (1.19–1.58)
*Study*: Lippman 2007 *Location*: Sao Paulo, Brazil *Population*: low-income women in Sao Paulo recruited from the clinic population and other non-clinical settings	*Intervention*: participants in the intervention group were given a kit to take home which included the xenostrip TV test and materials, a brochure with instructions and captioned pictures describing how to collect a vaginal sample and conduct and interpret the test. Also, a dacron swab used for self-collection to be returned to the clinic. Participants were instructed to test and collect within 14 days of enrolment and to return the specimen and test within 7 days of sample collection *Control*: women assigned to the control group received an indistinguishable kit, which included condoms, information regarding STIs, local resources for reproductive health services, and an appointment card for STI screening at the clinic. Clinic appointments were schedules within 2 weeks of enrolment	*Study design*: RCT *Sample size*: n=818 Intervention: n=410 Control: n=408 *Specimens tested*: vaginal swab *Diagnostic test*: Roche Cobas Amplicor CT/NG *Primary endpoint*: 2 weeks after enrolment	Lower proportion of women assigned to the intervention group tested for Ct, Ng and TV (393/410) compared with the control group (394/408) PR=0.99 (0.97–1.02) Lower proportion of CT cases in the intervention group (31/410) compared with the control group (35/408) PR=0.88 (0.55–1.40). Slightly lower proportion of Ng cases in the intervention (8/410) compared with the control group (8/408) PR=1.00 (0.38–2.63)
*Study*: Jones 2007 *Location*: Gugulethu, South Africa *Population*: young women (14–25) in a resource-poor area of Gugulethu, South Africa who were tested at two public health clinics	*Intervention*: women took home a kit consisting of a paper bag containing: two vaginal swabs in sealed plastic tubes; a xenostrip TV test with a small container of buffer solution; instructions on how to use the kit with diagrams, and a toll-free phone number; two self-administered questionnaires; an addressed envelope with prepaid postage for mailing materials. Home kits were collected from the post office daily *Control*: women in the clinic group received identical looking paper bag, containing condoms and educational materials and a clinic appointment card. At their appointment, women were given two swabs for self-sampling and the rapid TV test for self-testing. Nurses observed self-sampling and self-testing and recorded any difficulties the participant displayed	*Study design*: RCT *Sample size*: n=626 Intervention: n=313 Control: n=313 *Specimens tested*: vaginal swabs *Diagnostic test*: Roche Cobas Amplicor for CT/NG Primary endpoint: 6 weeks	Higher proportion of women assigned to the intervention group tested for Ct, Ng and TV (143/313) compared with the control group (132/313) PR=1.08 (0.91–1.29) Lower proportion of CT cases in the intervention group (21/313) compared with the control group (35/313) PR=0.60 (0.36–1.01). Higher proportion of Ng cases in the intervention (11/313) compared with the control group (10/313) PR=1.10 (0.47–2.55)
*Study*: Xu 2011 *Location*: STD and family planning clinics in New Orleans, LA; St. Louis, MO; and Jackson, MS; USA *Population*: low-income women infected with Ct attending STD clinics and family planning clinics in three US cities	*Intervention*: women were mailed or took home a specimen collection kit with instructions on how to obtain a vaginal swab for Ct diagnosis *Control*: women assigned to the control group attended a clinic for self-obtained vaginal swab for Ct diagnosis	*Study design*: RCT *Sample size*: STD clinic n=811, FP clinic n=412 Intervention: STD clinic n=408, FP clinic n=196 Control: STD clinic n=403, FP clinic n=208 *Specimens tested*: vaginal swab *Diagnostic test*: Aptima Combo 2 *Primary endpoint*: 7 weeks	Higher proportion of women assigned to the intervention group tested for Ct (STD clinic n=128/408, FP clinic n=96/196) compared with the control group (STD clinic n=101/403, FP clinic n=58/208) PR=STD clinic: 1.25 (1.00–1.56), FP clinic: 1.76 (1.35–2.28) Lower proportion of CT cases in the intervention group for STD clinics (17/408) compared with the control group (19/403) PR=0.88 (0.47–1.68). Higher proportion of CT cases in the intervention group for FP clinics (12/196) compared with the control group (8/208) PR=1.59 (0.66–3.81)
Study: Graseck 2010 Location: St. Louis, MO, USA *Population*: women (14–45) participating in the contraceptive CHOICE project to promote the use of long-acting reversible contraception in the St. Louis area	*Intervention*: a vaginal swab and collection tube were provided, identical to those used at baseline screening. Detailed step-by-step instructions with photographs explained how to collect the specimen and return it in a prepaid, preaddressed mailer *Control*: women randomised to the control arm were able to test with their regular healthcare provider or clinic or at four local family planning clinics. Women in the control were mailed written instructions, which stated that participants could be reimbursed for any expenses of STI testing and treatment. The clinics were provided with self-collected vaginal swab kits as used in the home-based group	*Study design*: RCT *Sample size*: n=558 Intervention: n=273 Control: n=285 *Specimens tested*: vaginal swabs *Diagnostic test*: BD ProbeTec *Primary endpoint*: 12 months	Higher proportion of women assigned to the intervention group tested for Ct (151/273) compared with the control group (92/285) PR=1.71 (1.40–2.09) Lower proportion of CT cases in the intervention group (3/273) compared with the control group (4/285) PR=0.78 (0.18–3.47)
*Study*: Reagan 2012 *Location*: St. Louis County, USA *Population*: men (18–45) residing in St. Louis County	*Intervention*: men were provided a sterile urine collection cup, NAAT urine transport tube, step-by-step instructions with photographs that explained how to collect and transfer the specimen. Each participant collected their own urine sample and transferred the sample to the transport tube. Instructions for returning the specimen were provided *Control*: men assigned to the control group were provided the same collection kit as in the intervention group to use in the clinic setting. The specimens were returned to the research staff	*Study design*: RCT *Sample size*: n=200 Intervention: n=100 Control: n=100 *Specimens tested*: urine *Diagnostic test*: BD ProbeTec *Primary endpoint*: 12 weeks post-enrolment	Higher proportion of men assigned to the intervention group tested for Ct and Ng (72/100) compared with the control group (48/100) PR=1.50 (1.18–1.90) Lower proportion of CT cases in the intervention group (1/100) compared with the control group (3/100) PR=0.33 (0.04–3.15). Higher proportion of Ng cases in the intervention group (3/100) compared with the control group (0/100) PR=7.00 (0.37–133.78)
*Study*: Gotz 2013 *Location*: Netherlands *Population*: heterosexual men and women at an STI clinic with a Ct infection	*Intervention*: a collection kit to the clinic group sent to their address of choice which could be sent back free of charge to the lab directly by mail *Control*: invitation to retest in the clinic was sent to the participants. Without appointment, participants were given a personal test kit at the waiting counter of the clinic to self-collect specimen for retesting	*Study design*: RCT *Sample size*: n=216 Intervention: n=109 Control: n=107 *Specimens tested*: men: urine, women: vaginal swabs *Diagnostic test*: BD Viper SDA *Primary endpoint*: 6 months	Higher proportion of people assigned to the intervention group tested for Ct (50/109) compared with the control group (25/107) PR=1.96 (1.32–2.93) High number of Ct cases diagnosed in the intervention group (8/109) compared with the control group (5/107) PR=1.63 (0.55–4.82)
*Study*: Klovstad 2013 *Location*: Rogaland, Norway *Population*: all men and women (18–25) registered in the national population register in Rogaland County, Norway	*Intervention*: participants were mailed a package to their home address consisting of a letter with information on chlamydia and the importance of testing and treatment and an invitation to take a home test free of charge, a urine container, a durable water-tight plastic container, instructions to obtain the first void urine sample and a prepaid return envelope and questionnaire *Control*: received no intervention and were not informed about the trial and thus continued the current strategy of testing in the healthcare system. Sample obtained in the healthcare system included either cervical or urethral swabs or first void urine	*Study design*: RCT *Sample size*: n=41 519 Intervention: n=10 000 Control: n=31 519 *Specimens tested*: home: urine, clinic: at least one urine sample, cervical or urethral swab *Diagnostic test*: BD ProbeTec CT DNA assay *Primary endpoint*: 4 months	Higher proportion of men and women assigned to the intervention group tested for Ct (men: 673/5077, women: 980/4923) compared with the control group (men: 229/16002, women: 843/15516) PR=men: 9.26 (8.00–10.73), women: 3.66 (3.36–3.99) Higher proportion of CT cases in the intervention group (men: 39/5077, women: 68/4923) compared with the control group (men: 44/16002, women: 81/15516) PR=men: 2.79 (1.82–4.29), women: 2.57 (1.86–3.55)
*Study*: Smith 2015 *Location*: Melbourne and Sydney, Australia *Population*: men and women 16 years and older with a recent Ct diagnosis	*Intervention*: 3 months after Ct diagnosis, an SMS was sent by the research team to let the patient know their retest was due and a kit would soon be mailed to them. The home collection kit contained the collection devices plus illustrated collection instructions, a laboratory request form, and a prepaid envelope. Mailing instructions were also included *Control*: 3 months after Ct diagnosis, patients were sent an SMS by the clinic to remind them to return to the clinic for retesting. This is routine practice at the two participating clinics. At one clinic, an opt-out system was used where the SMS was automatically generated on receipt of a positive result, and at the other, the automated SMS system was activated via the electronic patient management system by the attending clinician. Specimens collected at the clinics were tested by the usual pathology provider according to standard protocol	*Study design*: RCT *Sample size*: n=600 Intervention: n=302 Control: n=298 *Specimens tested*: women: vaginal swabs, men: urine, MSM: urine and rectal swabs *Diagnostic test*: BD ProbeTec ET CT and NG Amplified DNA assay, Roche Cobas 4800 CT/GC, Artus CT plus RG PCR kit *Primary endpoint*: 4 months	Higher proportion of men and women assigned to the intervention group tested for Ct (men: 57/100, women: 66/103, MSM: 61/98) compared with the control group (men: 34/99, women: 38/97, MSM: 45/102) PR=men: 1.64 (1.19–2.27), women: 1.64 (1.23–2.18), MSM: 1.41 (1.08–1.84) Higher proportion of CT cases in the intervention group (men: 7/101, women: 8/103, MSM: 16/98) compared with the control group (men: 5/99, women: 2/97, MSM: 5/102) PR=men: 1.37 (0.45–4.18), women: 3.77 (0.82–17.30), MSM: 3.33 (1.27–8.74)
*Study*: Kersaudy-Rahib 2015 *Location*: France *Population*: sexually active young men and women (18–24) in France	*Intervention*: the home self-collection kit included contact, consent form, instructions and materials. The sampling device included a urine collection device with three sponges for men and a dry self-taken vulvovaginal swab for women *Control*: the control group received tailored information to be screened in primary care	*Study design*: RCT *Sample size*: n=11 075 Intervention: n=5531 Control: n=5544 *Specimens tested*: urine *Diagnostic test*: Roche Cobas 4800 *Primary endpoint*: 6 weeks	Higher proportion of men and women assigned to the intervention group tested for Ct (men: 614/2573, women: 1002/2958) compared with the control group (men: 136/2579, women: 344/2965) PR=men: 4.53 (3.79–5.40), women: 2.92 (2.61–3.26) Higher proportion of CT cases in the intervention group (110/5531) compared with the control group (30/5544) PR=3.68 (2.46–5.49)
*Study*: Wilson 2017 *Location*: London, UK *Population*: men and women (16–30) living in the London boroughs of Lambeth and Southwark recruited in community settings	*Intervention*: participants in the home group were sent a text message with the URL to a site that offers free postal self-sampling test kits for Ct, Ng, HIV and syphilis. Participants who ordered a test kit from the website were required to complete a short order form. Those reporting STI symptoms were advised to a visit local clinic for immediate treatment. All test kits contained a lancet and collection tube to obtain a blood sample for syphilis and HIV. For Ct and Ng women were sent vaginal swabs, and men were sent first-catch urine and pharyngeal and rectal swabs. Pictorial leaflets with guidance on how to collect the specimen. A video demonstrating the blood collection was available on YouTube and could be accessed through the site. Test results delivered by text. Positive results were signposted to local clinics for confirmatory testing and treatment *Control*: participants in the control group were sent a URL of a bespoke website with the contact details and location of sexual health clinics in Lambeth and Southwark. These clinics provided usual care via walk-in services. Those diagnosed with an STI were asked to attend clinic for treatment	*Study design*: RCT *Sample size*: n=1739 Intervention: n=921 Control: n=818 *Specimens tested*: women: vaginal swabs, men: urine, pharyngeal and rectal swabs *Diagnostic test*: Cepheid GeneXpert Dual Target *Primary endpoint*: 6 weeks	Higher proportion of men and women assigned to the intervention group tested for STIs (men: 147/424, women: 289/604, MSM: 48/129) compared with the control group (men: 74/422, women: 99/609, MSM: 27/133) PR=men: 1.98 (1.55–2.53), women: 2.94 (2.41–3.59) MSM: 1.54 (1.21–1.95) Higher proportion of CT cases in the intervention group (12/921) compared with the control group (4/818) PR=1.17(0.38–3.59). Lower proportion of Ng cases in the intervention group (6/921) compared with the control group (5/818) PR=0.47 (0.14–1.51)

Ct, *Chlamydia trachomatis*; LCx, ligase chain reaction; MSM, men who have sex with men; Ng, *Neisseria gonorrhoeae*; PR, prevalence ratio; RCT, randomised controlled trial; SDA, strand displacement amplification; STIs, sexually transmitted infections.

### Intervention

The intervention was unsupervised, specimen self-collection outside a healthcare facility. Collection could be anywhere outside a traditional healthcare facility that would provide medical supervision, including inside the home. We refer to this mode of testing as specimen self-collection at home throughout the rest of the manuscript for increased readability and ease.

### Comparison

The comparison was specimen collection within a healthcare facility—either self-collected or HCP-collected for Ct and/or Ng testing or screening.

### Outcomes

#### Primary questions

1. Does specimen self-collection at home increase Ct and/or Ng testing uptake compared with collection (self or clinician) in clinical settings?
2. Are there any differences in the proportion of Ct and/or Ng infections detected between the intervention and control?
3. Are there any differences in the linkage to care for individuals who are positive for Ct and/or Ng between the intervention and control?
4. What is the concordance between specimen self-collection at home and specimen collection in clinical settings for the same individual?

#### Secondary questions

1. What are the harms/adverse effects associated with specimen self-collection at home for Ct and/or Ng testing?

To be included in the review, the study had to meet the following criteria. (a) Directly compared specimen self-collection at home or in other non-clinical settings to collection in a clinical setting (self or HCP) for Ct and/or Ng testing or screening. (b) Published in peer-reviewed journals in English. (c) Studies that used WHO-recommended Ct and Ng diagnostic assays. (d) Randomised controlled trials (RCTs) and cross-sectional studies that evaluated the concordance between specimens collected at home and in clinical settings. A full review protocol is available on PROSPERO (CRD42024466264).

### Search strategy and screening process

To identify relevant literature in an unbiased approach for our systematic literature review and meta-analysis, we requested a literature search from the Stephen B. Thacker CDC library. We initiated the request on 10 April 2023, by providing rationale for the study, inclusion/exclusion criteria, primary/secondary questions and any seed articles we identified on our own. The results of the request were returned on 19 April 2023. Databases included Medline (OVID), Embase (OVID), Global Health (OVID), Cochrane Library, CINAHL (EBSCOHost), Scopus and ClinicalTrials. Search terms included, but were not limited to home collection, self-collection, chlamydia, gonorrhoea and mail-in testing (online supplemental file 1).

To assist with review management and record keeping, we used the review management tool, Covidence. Two authors, PGT and ACS, independently assessed eligibility via an initial title and abstract screen followed by a full-text review. The screeners discussed any disagreements and consulted EK, when needed, for resolution.

### Data extraction and management

The authors developed and used a standardised data extraction form to extract data from all the included studies. To ensure the accuracy of extracted data, either PT or ACS extracted data while the other checked the information for accuracy.

### Assessing the risk of bias

To assess the risk of bias (RoB) in included RCTs, the authors used the Cochrane Risk of Bias (RoB2) tool. The RoB2 tool evaluates bias in five domains: randomisation process, deviations from intended interventions, missing outcome data, measurement of the outcome and selection of the reported result. The overall RoB for each study was determined based on the judgements made for each of the five domains. Each domain is assessed for RoB as either low, some concerns or high. The authors followed the guidelines provided by the Cochrane Handbook for Systematic Reviews of Interventions to assess the RoB in the included studies. Non-randomised cross-sectional studies were evaluated using the ROBINS-I tool which evaluates bias across seven domains. PT and ACS independently assessed the RoB in each study and any discrepancies were resolved through discussion. The results of the risk assessment were plotted in R Studio using the robvis (V.0.3.0.9) package.^37^

### Data analysis

To evaluate testing uptake and linkage to care at home in this meta-analysis, we calculated and reported pooled prevalence ratios (PRs) with 95% confidence intervals (CIs) using a random effects model using the package metafor^38^ in R Studio. To evaluate prevalence of Ct or Ng at home and in clinical settings we calculated and reported PRs with 95% CIs on an intent-to-treat basis. Heterogeneity among the studies was assessed using the I^2^ statistic. Subgroup analyses based on sex and specimen type were conducted to explore the sources of heterogeneity. For cross-sectional studies that compared the test results between specimen self-collection at home and collection in a clinical setting from the same individual, we did not pool the results. However, we calculated the concordance (per cent agreement) for each study and described the results in a narrative format. Harms and issues associated with self-collection at home were described in a narrative format, and no pooled effect estimate was calculated. Finally, publication bias was assessed visually using a funnel plot and statistically using Egger’s regression test.

### Quality of the evidence

We used the Grading of Recommendations, Assessment, Development and Evaluation (GRADE) approach to assess the quality of evidence based on the RoB, indirectness, inconsistency, imprecision and publication bias.

### Patient and public involvement

Patients and public were not directly involved in this review; we used publicly available data for the analysis.

## Results

**Figure 1:**
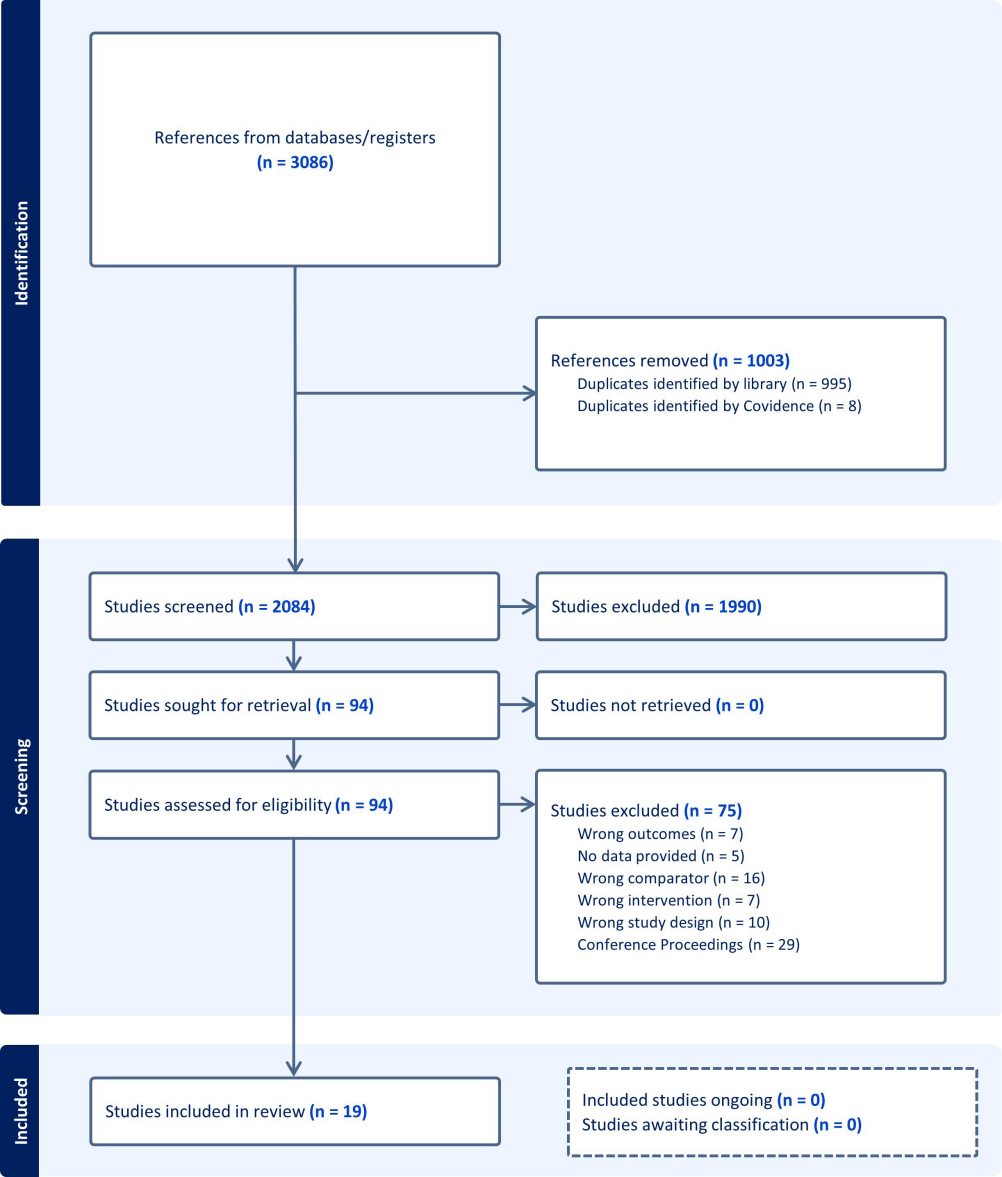
Preferred Reporting Items for Systematic Reviews and Meta-Analyses flowchart of the publication inclusion process.

A total of 3086 studies were assessed for title and abstract relevance (figure 1). After initial screening, 94 studies were deemed to have potential merit and selected for full-text review. During the full-text review phase, selected studies were thoroughly evaluated against the predefined inclusion and exclusion criteria. Following the full-text review, 75 studies were excluded from further consideration due to factors such as not addressing the primary outcomes, incorrect study design or studies that did not evaluate comparable specimen types between home and clinical settings (figure 1). After the initial search strategy, one paper that met inclusion criteria was identified and added on 1 June 2024. Ultimately, a total of 19 publications successfully met all inclusion criteria and were included for final analysis in this literature review and meta-analysis.

### Included studies

The present literature review and meta-analysis encompass a comprehensive analysis of 19 studies, comprising 4 concordance studies and 15 RCTs from 1998 to 2024. One of the included published reports contributed data from two separate RCTs (table 1). Among the RCTs, 13 incorporated female participants,^39^,^50^ and 8 included male participants^39^,^4249^; 2 studies specifically focused on MSM.^41 50^ The cumulative population size under investigation across all included RCTs was 62 369 individuals. These studies were conducted across various global regions, with five RCTs originating from the USA,^47 48 52 52^ three from Denmark^42 43 51^ and one each from the UK,^41^ Australia,^50^ France,^40^ the Netherlands,^39^ Norway,^49^ South Africa^44^ and Brazil.^46^ Within the RCTs, three studies primarily evaluated the retesting of individuals previously diagnosed with the infection.^39 45 50^ Two studies examined testing uptake in partners of index patients who had tested positive for either Ct or Ng.^43 51^ All but one of the RCTs focused on Ct testing,^39^,^4446^ while five studies specifically investigated Ng testing.^41 44 46 47 52^ Furthermore, RCTs assessed the proportion of individuals who tested positive and subsequently received appropriate treatment.^41 44 46 49^

From the four cross-sectional concordance studies, two were conducted in Canada,^53 54^ and one each from the USA^55^ and Belgium.^56^ All these studies had the same participants collect the same type of specimen at home and in a clinical setting. These studies had a total of 906 participants; three studies exclusively included MSM^54^,^56^ and one exclusively included women.^53^ All studies evaluated concordance for both Ct and Ng infections, but each evaluated different specimen types.

### Uptake of STI testing at home and in clinical settings

**Figure 2:**
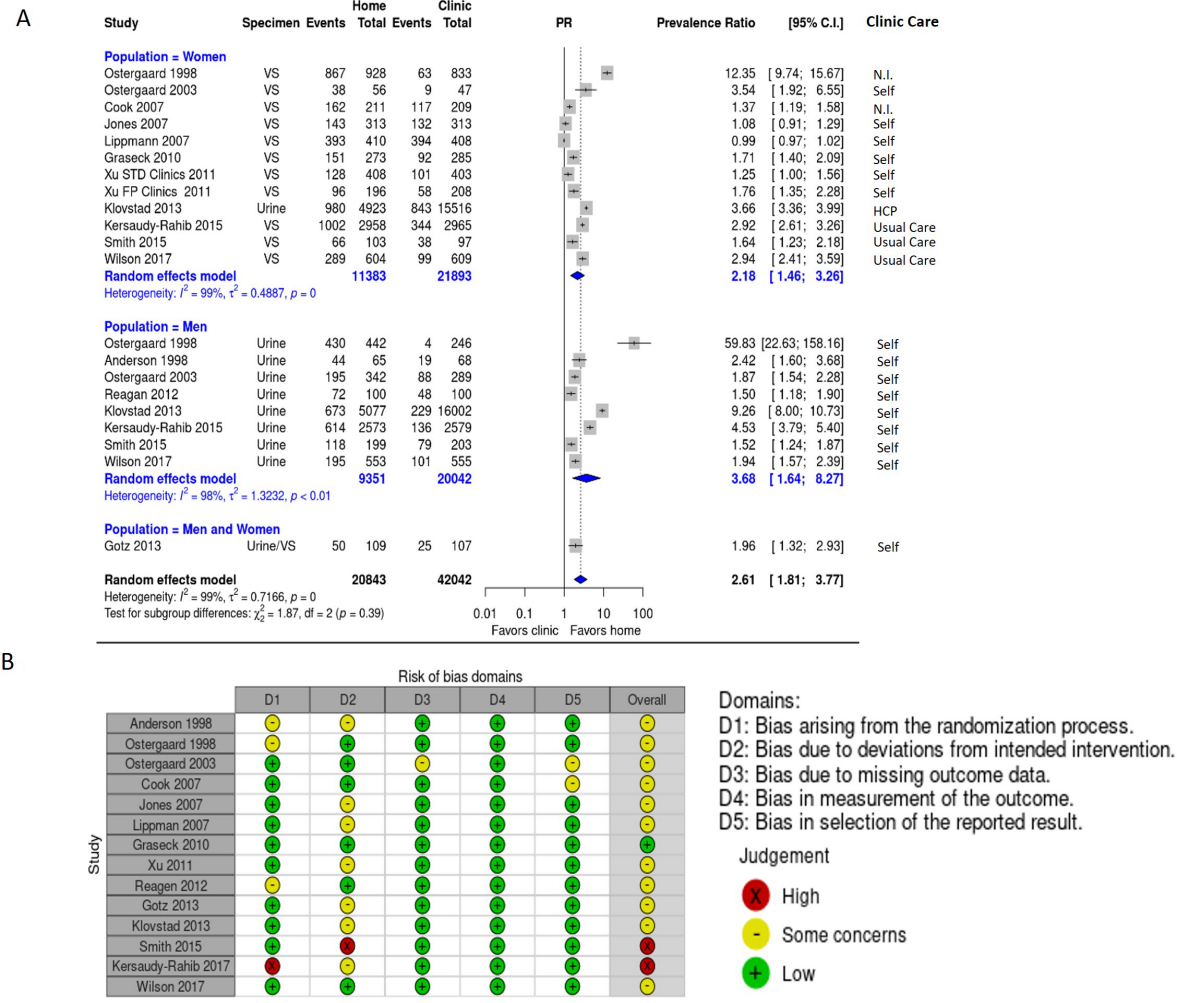
Uptake of Ct/Ng testing in sexually active persons. Forest plot depicting the effect of specimen self-collection at home compared with specimen collection in clinical settings for Ct and Ng testing. Clinic care represents who collected the specimen, either HCP, self, no information (N.I.) or usual care was indicated. (**B**) Risk of bias for studies that investigated uptake of testing at home and in clinical settings. Green=low risk of bias, yellow=some concerns, red=high risk of bias. Ct, *Chlamydia trachomatis*; FP, family planning; Ng, *Neisseria gonorrhoeae*; PR, prevalence ratio; STD, sexually transmitted disease; VS*,* vaginal specimen.

Fifteen RCTs measured the uptake of STI testing for those randomised to specimen self-collection at home in comparison to specimen collection in clinical settings.^39^,^52^ Overall, we found that significantly more men and women collected and returned specimens for Ct and/or Ng testing at home compared with those randomised to clinical settings (PR=2.61 (95% CI 1.81–3.77), I^2^=99%, figure 2A). Due to the high heterogeneity, we also investigated subgroup differences between men and women. There was no significant difference between men and women when evaluating uptake of testing (p=0.26); indicating this was not the cause of the high heterogeneity observed among the studies. However, men were over 3.5 times as likely to get tested for Ct or Ng at home compared with clinical settings (PR=3.68 (95% CI 1.64–8.27), I^2^=99%) while women were only over twice as likely to get tested for Ct or Ng (PR=2.18 (95% CI 1.46–3.26), I^2^=99%). Despite the overall high heterogeneity measured by the I^2^ statistic, all but two studies demonstrated significantly more Ct/Ng testing for those randomised to specimen self-collection at home. For all studies that evaluated uptake of testing, we evaluated the RoB using the Cochrane RoB2 criteria. Most of the studies had a low RoB in all categories, while a few studies had some serious concerns relating to the randomisation process and adherence to the intervention (figure 2B).

### Proportion of positive Ct and Ng infections detected

14 RCTs reported the number of positive Ct tests.^39^,^4446^ Meta-analysis of these RCTs suggested a significantly greater proportion of positive Ct tests in the individuals who collected specimens at home compared with the specimens collected in clinical settings with an overall PR of 1.61 (95% CI 1.10–2.35) (online supplemental figure 1A). Only five of the included RCTs provided data regarding the number of positive Ng tests detected.^41 44 46 47 52^ Moreover, there was a relatively limited number of infections detected overall, with 28 out of 2127 testing positive in the intervention group and 24 out of 2138 testing positive in the control group. The analysis of Ng tests did not reveal a significant difference in the proportion of infections detected between the intervention (at home) and the control (clinical settings) groups (PR=1.16 (95% CI 0.68–1.97), online supplemental figure 1B). We also evaluated the RoB for the proportion of positive Ct or Ng tests. Most studies demonstrated low RoB across all outcomes, but a few studies had some concerns and serious concerns for bias in the randomisation process, including deviations from the intended intervention and bias in the measurement of the outcome.

### Linkage to care

**Figure 3:**
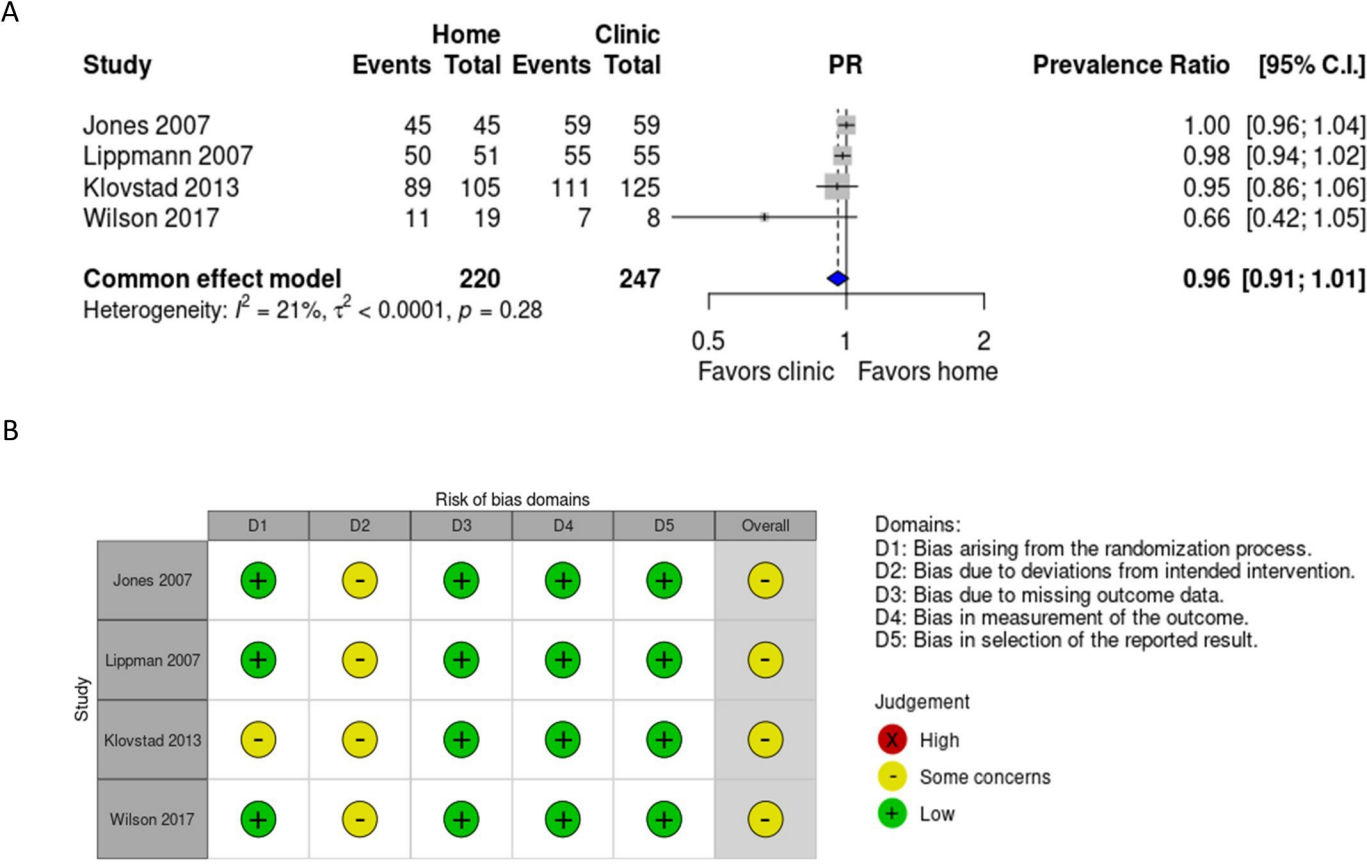
Proportion of *Chlamydia trachomatis*/*Neisseria gonorrhoeae* infections treated. Forest plot depicting the effect of specimen self-collection at home compared with specimen collection in clinical settings on the proportion of infections treated. (**B**) Risk of bias for studies that investigated the number of infections treated for those diagnosed at home and in clinical settings. Green=low risk of bias, yellow=some concerns. PR, prevalence ratio.

Of the included RCTs, four provided information about treatment following positive Ct or Ng test results.^41 44 46 49^ Within those four studies, we found no significant difference in the proportion of treated cases between the intervention and control groups (PR=0.96 (95% CI 0.91–1.01), figure 3A), with 88.6% (195/220) of the intervention group treated compared with 93.9% (232/247) treated in the comparison group. The quality of evidence was high, suggesting that the use of specimen self-collection at home for STI testing provides appropriate linkage to treatment.

### Test concordance between self-collection in clinical settings and home collection

Four studies evaluated the test concordance (per cent agreement) for specimens collected at home compared with specimens collected in clinical settings by the same individual.^53 55 56^ Due to the small number of studies, and lack of overlap between anatomic sites, we did not pool any of the following results. In one study, conducted in Canada with 198 women aged 16–25, the Ct and Ng result concordance between the specimens collected at home and clinical settings was assessed for vaginal swabs, urine and these two specimens pooled together.^53^ Women who had not previously self-collected vaginal swabs were assigned to home collection first and were given a kit with two swabs and two tubes for vaginal self-collection and a urine collection jar.^53^ The order of collection of vaginal swabs and urine was randomised in both settings. Sampling in both settings was performed on the same day or within the week. Samples were transferred to the laboratory and tested within 24 hours for Ct and Ng using the Aptima Combo 2 assay on a Panther Instrument (Hologic, Inc). Specimen self-collection in the clinic and self-collection at home resulted in high test result concordance for all specimen types assessed. For Ct, concordance was 96.7% for vaginal swabs, 96.5% for urine and 96.3% for pooled specimens (table 2). The highest prevalence was observed using vaginal swabs for both Ct (15.2% home, 14.6% clinic) and Ng (6.6% home, 6.1% clinic). The lowest prevalence was observed using urine for both Ct (11.1% home, 11.6% clinic) and Ng (5.6% home and clinic). For Ct, one positive case was missed for at-home sampling for both urine and pooled specimens compared with clinical self-collection. One additional positive Ct case was detected using vaginal swabs collected at home compared with vaginal swabs collected in the clinic. For Ng, concordance was 92.3% for vaginal swabs and 100% for urine and pooled specimens (table 2). One additional positive Ng case was detected with vaginal swabs collected at home compared with vaginal swabs collected in the clinic.

**Table 2:** Concordance between specimen self-collection at home and in clinical settings

Study	Specimen	Ct/Ng	Concordance (%)	TP	TN	FP	FN	Total specimens
Salow 2017	Rectal swab	Ct	95.0	11	123	0	7	141
	Pharyngeal swab	Ct	99.3	1	143	1	0	145
	Rectal swab	Ng	95.7	12	123	0	6	141
	Pharyngeal swab	Ng	97.2	11	131	0	3	145
De Baetselier 2019	Urine	Ct	99.1	6	454	3	1	464
	Urine	Ng	99.6	7	455	2	0	464
Chernesky 2019	Urine	Ct	96.5	22	175	0	1	198
	Vaginal swab	Ct	96.7	29	169	1	0	198
	Pooled	Ct	96.3	26	172	0	1	198
	Urine	Ng	100	11	187	0	0	198
	Vaginal swab	Ng	92.3	12	186	1	0	198
	Pooled	Ng	100	12	186	0	0	198
Orser 2024	Rectal swab	Ct	96.0	23	195	4	5	227
	Pharyngeal swab	Ct	99.0	5	281	2	1	289
	Rectal swab	Ng	100	11	216	0	0	227
	Pharyngeal swab	Ng	99.7	17	271	1	0	289

Concordance (per cent agreement) was calculated within the same specimen type for home and clinical collection. ‘Total specimens’ refers to the total number of paired specimens received.

Ct, *Chlamydia trachomatis*; Ng, *Neisseria gonorrhoeae*.

Three studies evaluated the concordance between specimens self-collected at home and in clinical settings among MSM.^54^,^56^ In a study by De Baetselier *et al* in Belgium,^56^ MSM on PrEP collected urine at home using a standardised device and mailed it to a lab for testing using the Abbot CT/NG RT assay, then collected another urine sample at a clinic. Across 471 at-home specimens with matching clinic samples, there was high concordance for Ct (99.1%) and Ng (99.6%). One positive Ct sample was missed for at-home specimen collection, but three additional Ct cases were detected through at-home collection. For Ng, two additional cases were detected in the samples collected at home.

Two studies assessed concordance for extragenital specimens in the USA^55^ and Canada.^54^ In the USA study, clinic-based rectal swabs were self-collected, and pharyngeal swabs were collected by research associates and placed in the Xpert Vaginal/Endocervical Specimen Collection Kit. Extragenital specimens collected at home were mailed in within 48 hours of a clinic visit. For symptomatic individuals, they collected both sets of pharyngeal and rectal samples within the clinic to ensure treatment did not impact results. Specimens collected at home had a high concordance with those collected at the clinic (Ct: 95.0% rectal, 99.3% pharyngeal; Ng: 95.7% rectal, 97.2% pharyngeal). However, home collection missed 28% (N=9/32) of Ng infections and 37% (7/19) of Ct infections. In the Canadian study, MSM 16 years or older self-collected pharyngeal and rectal specimens at home and brought the specimens into the clinic where they were swabbed again, either by an HCP or self, depending on patient preference. Both specimens collected at home and in the clinic were tested using the Roche Cobas CT/NG assay on the Cobas 8800 system. Positive Ng results were confirmed using the PivNG assay V2 on the Roche Cobas omni utility channel. Concordance was high between the two settings (Ct: 96.0% rectal, 99.0% pharyngeal; Ng: 100% rectal, 99.7% pharyngeal). Home collection identified six Ct infections that were missed by specimens collected in the clinic. Of the results that were positive from clinic collection but not from the specimens collected at home, a majority had cycle threshold values higher than 35, indicating a low amount of bacterial DNA present in the specimen. Together, these results suggest that collection at home and in clinical settings are highly concordant, but some cases are potentially missed with collection in either setting.

### Harms associated with at-home specimen self-collection

Only three of the included RCTs investigated potential harms associated with specimen self-collection at home versus collection in clinical settings.^44 47 57^ More people in the home specimen collection group reported some level of difficulty understanding the instructions for the kit compared with those in a clinical setting group.^47^ Importantly, 2.6%–17% of individuals randomised to the home self-collection group reported feeling pain or discomfort during specimen self-collection, compared with 12.3% of the clinical self-collection group.^44 57^ Finally, in one of our included studies^55^ there was a slightly higher proportion of specimens collected at home with invalid results (1.7%) compared with those collected in clinical settings (0.5%).

## Discussion

### Summary

In this comprehensive literature review, we identified 19 studies, encompassing 15 RCTs involving a substantial cohort of 62 369 participants, alongside four cross-sectional studies. Our primary objectives were to evaluate if specimen self-collection at home and other non-clinical settings should be offered as an additional approach to STI testing based on testing uptake, prevalence of Ct or Ng, linkage to care, and the per cent agreement with collection in clinical settings. Offering specimen self-collection at home resulted in a significantly higher number of Ct and Ng tests, particularly among men which may be due in part to men in general are less likely to seek healthcare than women.^58^ A higher proportion of positive Ct cases was observed in specimens collected at home, likely due to asymptomatic individuals finding testing more convenient when provided outside clinics. There was no significant difference in the proportion of positive Ng cases between the intervention and control which might be due to the more pronounced symptoms in Ng infections compared with Ct infections^59^ and lower overall prevalence of Ng. Linkage to care was similar between specimens collected at home and in clinical settings. The four concordance studies showed high agreement in Ct and Ng results between home and clinical settings for self-collected vaginal swabs, urine and extragenital specimens.

### Consistency

Our results for testing uptake at home are consistent with previously published meta-analyses.^24 35 36^ All three meta-analyses reported increased uptake in Ct/Ng for those assigned to self-collect at home. We identified and included three additional studies^40 41 50^ that reported on index patient uptake and one additional study that evaluated retesting behaviours.^39^ One previous meta-analysis demonstrated there was an increased likelihood of a positive Ct test at home across four studies,^24^ which we also observed here (online supplemental figure 1). However, previous reviews limited the analysis to individuals who tested rather than all people who were randomised.^24 36^ This type of analysis resulted in a significant decrease in positive STI tests at home compared with those who had specimens collected in clinical settings. This should be interpreted with caution as symptomatic people, who have the highest retest probability, may be more likely to choose to test in the clinical setting and expect to receive immediate treatment.^45 50 60^

### Strengths

The inclusion of both RCTs and cross-sectional studies broadened the scope of our investigation, allowing for a more holistic assessment of self-collection for STI testing at home. This diverse selection of study designs enhances the validity and applicability of our findings and is balanced by our strict inclusion criteria and use of only peer-reviewed studies. The rigorous methodology employed for article selection, guided by a trained librarian, ensured the systematic identification of relevant studies, minimising the RoB in our dataset. Finally, the deliberate inclusion of studies from various socioeconomic contexts and encompassing populations with varied risk profiles, enhances the generalisability of our results and makes them relevant to a broader spectrum of healthcare settings and individuals. This diversity strengthens the depth and breadth of our discussion and the utility of our findings in informing public health strategies.

### Limitations

Some limitations exist. Data on the use of self-collected extragenital (rectal and pharyngeal) specimens at home for both men and women are scant. Only one RCT provided rectal swabs along with urine for MSM (36), and two cross-sectional studies evaluated the concordance of rectal and throat swabs in MSM collected at home compared with these same specimen types collected in the clinic.^54 55^ In the US study, the missed cases were likely due to the difference in transportation method as specimens collected at home used dry swabs that were reconstituted at the lab, while specimens collected in the clinic were transported in a liquid transport reagent. This highlights an ongoing need to understand how specimen transportation impacts the specimen stability and test sensitivity. Despite the limited data included in our literature review, self-collection of extragenital specimens is acceptable to patients and often preferred.^20 61^ The often asymptomatic nature of rectal and pharyngeal infection means screening is an important pathway to interrupt disease transmission that cannot be ignored.^62^,^64^ However, more research is needed to understand the feasibility and accuracy of extragenital specimen collection at home. Additionally, a key gap is a lack of studies conducted during or after the COVID-19 pandemic, which has undoubtedly increased the acceptability of many telehealth models involving self-testing.^65 66^ It will be important to understand how the pandemic influenced acceptability and feasibility of self-collection at home for STI testing. Finally, information regarding uptake and positivity for transgender individuals is severely lacking, and this key population needs to be included in future research to appropriately provide guidance.

Our comprehensive review and meta-analysis present a nuanced but encouraging perspective on the use of self-collection at home for Ct and Ng testing. According to GRADE, this review provides modest evidence that offering specimen self-collection for Ct and Ng testing at home leads to increased uptake among both men and women across diverse settings, without loss to follow-up or treatment. While internet-based testing is a strategy already used in some countries such as the UK^32^ and Australia,^33^ it is a novel strategy in Canada^54^ and the USA (FDA Grants Marketing Authorisation of First Test for Chlamydia and Gonorrhoea with at-home Sample Collection | FDA). Specimen self-collection at home could provide a valuable strategy to increase partner testing and retesting as a way to reach those who need it most. This approach may increase health equity by addressing critical barriers to screening, allowing broader uptake and enabling the identification and treatment of more asymptomatic individuals who might unknowingly contribute to disease transmission. Notably, our findings highlight that high concordance with specimens collected in clinical settings can be achieved. As we look ahead, future research should expand to include transgender individuals, assess linkage to care and fully explore the viability of collecting extragenital specimens at home.

While specimen self-collection at home for STI testing has high acceptability and reliability, several concerns need to be considered. Specimen self-collection at-home kits need to be appropriately priced to ensure accessibility. Currently available collection kits in the USA for Ct and Ng can be hundreds of US dollars,^31^ which is prohibitive for many. The need for a mailing address and internet access may also be limiting to some. Additionally, for those living with others, privacy may be a concern. Allowing patients to pick up and drop off specimen self-collection kits in healthcare settings could help address some of these key issues. Working within a healthcare setting would also ensure the proper tests are ordered and linkage to care and communication of results is done in an appropriate and timely manner. Community pharmacies are well-positioned for this need as they are typically open longer hours and are often conveniently located. Finally, the Faculty of Sexual and Reproductive Healthcare and the British Association for Sexual Health and HIV in the UK have developed standards for web-based sexual and reproductive health services.^18^ These standards guide providers and help users understand what to expect from online service providers and the development of similar standards should be considered for implementation elsewhere.

## Supplementary material

10.1136/bmjgh-2024-015349 online supplemental figure 1

10.1136/bmjgh-2024-015349 online supplemental file 1

## Data Availability

All data relevant to the study are included in the article or uploaded as supplementary information.
