# Supplementary figures and images for: At-home specimen self-collection as an additional testing strategy for chlamydia and gonorrhoea: a systematic literature review and meta-analysis
Source: BMJ Glob Health. 2024 Aug 27;9(8):e015349. doi: 10.1136/bmjgh-2024-015349 (PMC11404247; doi:10.1136/bmjgh-2024-015349)

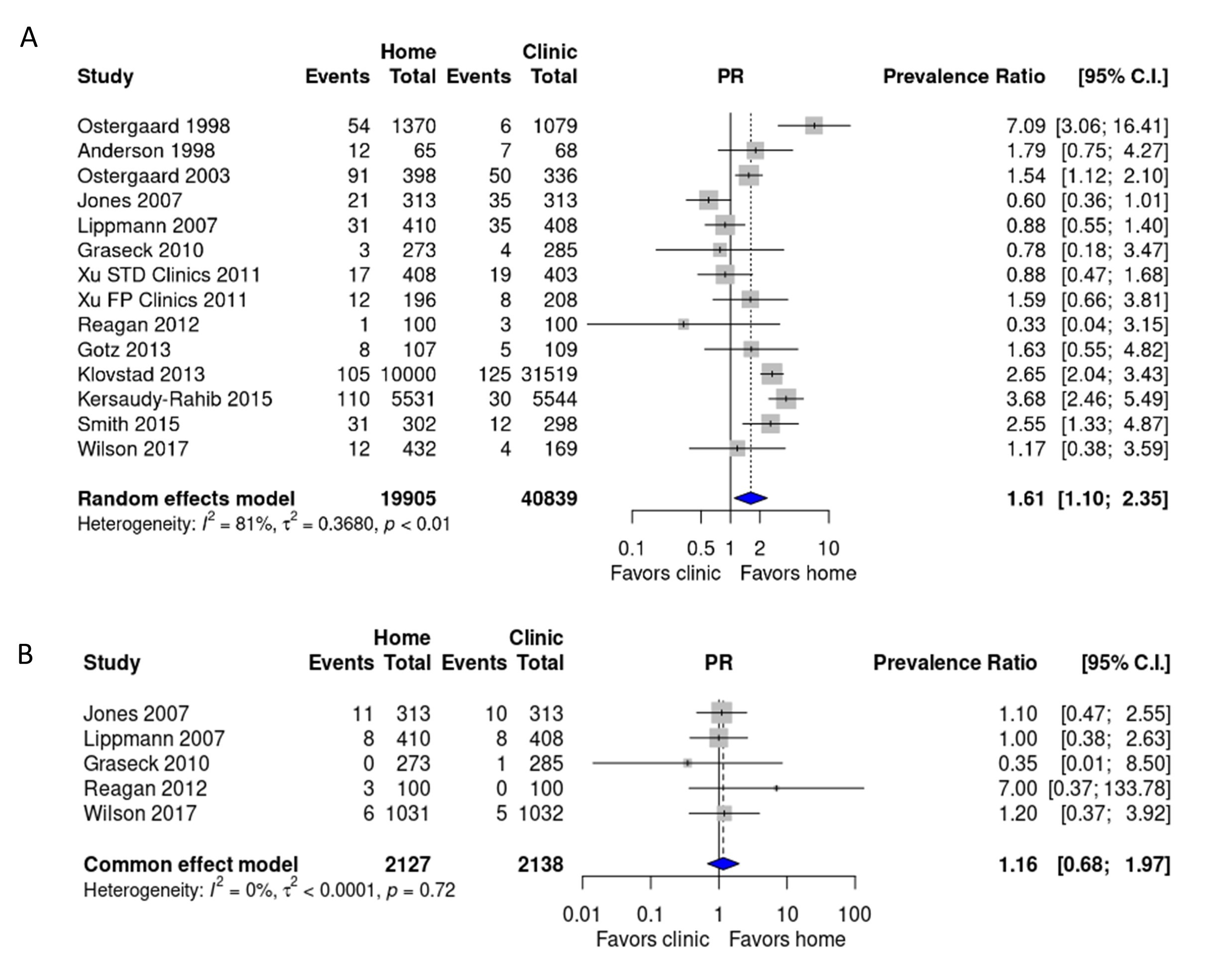

Supplement: online supplemental figure 1 [file bmjgh-9-8-s001.jpg]
