# Supplementary material for: At-home specimen self-collection as an additional testing strategy for chlamydia and gonorrhoea: a systematic literature review and meta-analysis
Source: BMJ Glob Health. 2024 Aug 27;9(8):e015349. doi: 10.1136/bmjgh-2024-015349 (PMC11404247; doi:10.1136/bmjgh-2024-015349)
Supplement: online supplemental file 1 [file bmjgh-9-8-s002.pdf]

**Requested by: Smith Amanda**

**Search Query:** systematic literature review to assess whether at-home self-collection for NG and CT is not inferior to collection in a healthcare facility and increases testing uptake.

**Primary questions:**

1. Does home self-collection increase CT and/or NG testing compared to collection in a healthcare facility?
2. What are the harms/ adverse effects associated with home specimen self-collection for CT and/or NG?
3. Are there any issues associated with home self-collection including the quality of specimen and the sensitivity/specificity compared to collection in a healthcare setting?

**Secondary questions:**

1. Does home self-collection lead to diagnosis of more CT and/or NG cases compared to collection in a healthcare facility?
2. Are there any differences in the number of treated individuals who are positive for CT and/or NG between the two collection groups?
3. Are there any barriers to self-collection at home that are not present in collection in a healthcare facility?
4. What is the acceptability and ease of home self-collection for NG and/or CT testing?

**Search Strategy:**

| Database | Strategy | Run Date | Records |
|---|---|---|---|
| **Medline (OVID) 1946-** | 1. Exp Chlamydia Infections/ OR exp Chlamydia/<br>2. Gonorrhea/ OR Neisseria gonorrhoeae/<br>3. Chlamydia*.ti,ab,kf,hw.<br>4. Gonorrh*.ti,ab,kf,hw.<br>5. OR/1-4<br>6. Mass Screening/mt OR Specimen Handling/ OR Urine Specimen Collection/ OR Diagnostic Tests, Routine/ OR Reagent Kits, Diagnostic/<br>7. Self Care/ OR Home Care Services/ OR Postal Service/ OR exp Internet/<br>8. 6 AND 7<br>9. (home* ADJ5 test*) OR (home* ADJ5 screen*) OR (at-home* ADJ5 test*) OR (at-home* ADJ5 screen*) OR (self* ADJ5 test*) OR (self* ADJ5 screen*) OR (home* ADJ5 collect*) OR (at-home* ADJ5 collect*) OR (self* ADJ5 collect*) OR (home* ADJ5 sampl*) OR (at-home* ADJ5 sampl*) OR (self* ADJ5 sampl*) OR self-administ* OR (postal ADJ5 test*) OR (mail-in ADJ5 test*) OR (postal ADJ5 collection*) OR (postal ADJ5 specimen*) OR (mail-in ADJ5 | 04/19/2023 | 986 |

| | | | |
|---|---|---|---|
| | collect*) OR (mail-in ADJ5 specimen*) OR IWantTheKit OR (order* ADJ5 test*) OR (test* ADJ5 online)<br>10. 8 OR 9<br>11. 5 AND 10<br>12. limit 12 to english language | | |
| **Embase (OVID) 1974-** | 1. Exp Chlamydia Infection/ OR exp Chlamydia/<br>2. Gonorrhea/ OR Neisseria gonorrhoeae/<br>3. Chlamydia*.ti,ab,kf,hw.<br>4. Gonorrh*.ti,ab,kf,hw.<br>5. OR/1-4<br>6. Mass Screening/ OR Specimen Handling/ OR Urine Sampling/ OR Diagnostic Tests/ OR Diagnostic kit/<br>7. Self Care/ OR Home Care/ OR Postal mail/ OR exp Internet/<br>8. 6 AND 7<br>9. (home* ADJ5 test*) OR (home* ADJ5 screen*) OR (at-home* ADJ5 test*) OR (at-home* ADJ5 screen*) OR (self* ADJ5 test*) OR (self* ADJ5 screen*) OR (home* ADJ5 collect*) OR (at-home* ADJ5 collect*) OR (self* ADJ5 collect*) OR (home* ADJ5 sampl*) OR (at-home* ADJ5 sampl*) OR (self* ADJ5 sampl*) OR self-administ* OR (postal ADJ5 test*) OR (mail-in ADJ5 test*) OR (postal ADJ5 collection*) OR (postal ADJ5 specimen*) OR (mail-in ADJ5 collect*) OR (mail-in ADJ5 specimen*) OR IWantTheKit OR (order* ADJ5 test*) OR (test* ADJ5 online)<br>10. 8 OR 9<br>11. 5 AND 10<br>12. limit 12 to english language<br>13. limit 12 to "remove medline records" | 04/19/2023 | 669<br><br>- duplicates<br><br>= 609 unique items |
| **Global Health (OVID) 1910-** | 1. Chlamydia*.ti,ab,sh,id.<br>2. Gonorrh*.ti,ab,sh,id.<br>3. OR/1-2<br>4. (home* ADJ5 test*) OR (home* ADJ5 screen*) OR (at-home* ADJ5 test*) OR (at-home* ADJ5 screen*) OR (self* ADJ5 test*) OR (self* ADJ5 screen*) OR (home* ADJ5 collect*) OR (at-home* ADJ5 collect*) OR (self* ADJ5 collect*) OR (home* ADJ5 sampl*) OR (at-home* ADJ5 sampl*) OR (self* ADJ5 sampl*) OR self-administ* OR (postal ADJ5 test*) OR (mail-in ADJ5 test*) OR (postal ADJ5 collection*) OR (postal ADJ5 specimen*) OR (mail-in ADJ5 collect*) OR (mail-in ADJ5 specimen*) OR IWantTheKit OR (order* ADJ5 test*) OR (test* ADJ5 online) | 04/19/2023 | 701<br>- duplicates<br><br>= 73 unique items |

| | | | |
|---|---|---|---|
| | 5. 3 AND 4<br>6. limit 6 to english language | | |
| **Cochrane Library** | #1 ([mh "Chlamydia Infections"] OR [mh Chlamydia]) OR ([mh ^Gonorrhea] OR [mh ^"Neisseria gonorrhoeae"]) OR (Chlamydia*:ti,ab,kw) OR ((Gonorrh*:ti,ab,kf))<br>#2 (([mh ^"Mass Screening"] OR [mh ^"Specimen Handling"] OR [mh ^"Urine Specimen Collection"] OR [mh ^"Diagnostic Tests, Routine"] OR [mh ^"Reagent Kits, Diagnostic"]) AND ([mh ^"Self Care"] OR [mh ^"Home Care Services"] OR [mh ^"Postal Service"] OR [mh Internet])) OR ((home* NEAR/5 test*):ti,ab OR (home* NEAR/5 screen*):ti,ab OR ("at-home" NEAR/5 test*):ti,ab OR ("at-home" NEAR/5 screen*):ti,ab OR (self* NEAR/5 test*):ti,ab OR (self* NEAR/5 screen*):ti,ab OR (home* NEAR/5 collect*):ti,ab OR ("at-home" NEAR/5 collect*):ti,ab OR (self* NEAR/5 collect*):ti,ab OR (home* NEAR/5 sampl*):ti,ab OR ("at-home" NEAR/5 sampl*):ti,ab OR (self* NEAR/5 sampl*):ti,ab OR "self-administered":ti,ab OR (postal NEAR/5 test*):ti,ab OR (mail-in NEAR/5 test*):ti,ab OR (postal NEAR/5 collection*):ti,ab OR (postal NEAR/5 specimen*):ti,ab OR ("mail-in" NEAR/5 collect*):ti,ab OR ("mail-in" NEAR/5 specimen*):ti,ab OR IWantTheKit:ti,ab OR (order* NEAR/5 test*):ti,ab OR (test* NEAR/5 online):ti,ab)<br>#3 #1 AND #2 | 04/19/2023 | 198<br>- duplicates<br>=68 unique items |
| **CINAHL (EBSCOHost)** | S1 ((MH "Chlamydia Infections+") OR (MH Chlamydia+)) OR ((MH Gonorrhea) OR (MH "Neisseria gonorrhoeae")) OR ((TI Chlamydia* OR AB Chlamydia* OR SU Chlamydia*)) OR ((TI Gonorrh* OR AB Gonorrh* OR SU Gonorrh*))<br>S2 (((MH "Mass Screening") OR (MH "Specimen Handling") OR (MH "Urine Specimen Collection") OR (MH "Diagnostic Tests, Routine") OR (MH "Reagent Kits, Diagnostic")) AND ((MH "Self Care") OR (MH "Home Care Services") OR (MH "Postal Service") OR (MH Internet+))) OR ((home* N5 test*) OR (home* N5 screen*) OR (at-home* N5 test*) OR (at-home* N5 screen*) OR (self* N5 test*) OR (self* N5 screen*) OR (home* N5 collect*) OR (at-home* N5 collect*) OR (self* N5 collect*) OR (home* N5 sampl*) OR (at-home* N5 sampl*) OR (self* N5 sampl*) OR self-administ* OR (postal N5 test*) OR (mail-in N5 test*) OR (postal N5 collection*) OR (postal N5 specimen*) OR (mail-in N5 collect*) OR (mail-in N5 specimen*) OR IWantTheKit OR (order* N5 test*) OR (test* N5 online))<br>**Limiters** - English Language; Exclude MEDLINE records | 04/19/2023 | 90<br>- duplicates<br>=33 unique items |

| | | | |
|---|---|---|---|
| **Scopus** | ((INDEXTERMS("Chlamydia Infections") OR INDEXTERMS(Chlamydia)) OR (INDEXTERMS(Gonorrhea) OR INDEXTERMS("Neisseria gonorrhoeae")) OR (TITLE-ABS-KEY(Chlamydia*)) OR (TITLE-ABS-KEY(Gonorrh*))) AND (((INDEXTERMS("Mass Screening") OR INDEXTERMS("Specimen Handling") OR INDEXTERMS("Urine Specimen Collection") OR INDEXTERMS("Diagnostic Tests, Routine") OR INDEXTERMS("Reagent Kits, Diagnostic")) AND (INDEXTERMS("Self Care") OR INDEXTERMS("Home Care Services") OR INDEXTERMS("Postal Service") OR INDEXTERMS(Internet))) OR ((home* W/5 test*) OR (home* W/5 screen*) OR (at-home* W/5 test*) OR (at-home* W/5 screen*) OR (self* W/5 test*) OR (self* W/5 screen*) OR (home* W/5 collect*) OR (at-home* W/5 collect*) OR (self* W/5 collect*) OR (home* W/5 sampl*) OR (at-home* W/5 sampl*) OR (self* W/5 sampl*) OR self-administ* OR (postal W/5 test*) OR (mail-in W/5 test*) OR (postal W/5 collection*) OR (postal W/5 specimen*) OR (mail-in W/5 collect*) OR (mail-in W/5 specimen*) OR IWantTheKit OR (order* W/5 test*) OR (test* W/5 online))) AND NOT INDEX(medline)<br>"limit to english language" | 04/19/2023 | 389<br><br>- duplicates<br><br>=269 unique items |
| **Clinicaltrials** | Chlamydia OR Gonorrhea OR Neisseria gonorrhoeae<br>AND<br>Home OR self | 04/19/2023 | 55 |

Notes: Duplicates were identified using the Endnote automated "find duplicates" function with preference set to match on title, author and year, and removed from your Endnote library. There will likely be additional duplicates found that Endnote was unable to detect.
